# Iatrogenic Cerebral Amyloid Angiopathy Post Neurosurgery: Frequency, Clinical Profile, Radiological Features, and Outcome

**DOI:** 10.1161/STROKEAHA.122.041690

**Published:** 2023-04-10

**Authors:** Kanishk Kaushik, Ellis S. van Etten, Bob Siegerink, L. Jaap Kappelle, Afina W. Lemstra, Floris H.B.M. Schreuder, Catharina J.M. Klijn, Wilco C. Peul, Gisela M. Terwindt, Marianne A.A. van Walderveen, Marieke J.H. Wermer

**Affiliations:** 1 Department of Neurology (K.K., E.S.v.E., G.M.T., M.J.H.W.), Leiden University Medical Center, the Netherlands.; 2 Department of Clinical Epidemiology (B.S.), Leiden University Medical Center, the Netherlands.; 3 Department of Radiology (M.A.A.v.W.), Leiden University Medical Center, the Netherlands.; 4 Department of Neurology, University Medical Center Utrecht, the Netherlands (L.J.K.).; 5 Department of Neurology, Alzheimer center Amsterdam, Amsterdam University Medical Center, the Netherlands (A.W.L.).; 6 Department of Neurology, Donders Institute for Brain, Cognition and Behavior, Radboud University Medical Center, the Netherlands (F.H.B.M.S., C.J.M.K.).; 7 University Neurosurgical Center Holland, LUMC|HMC|HAGA Leiden & The Hague, the Netherlands (W.C.P.).

**Keywords:** amyloid, cerebral amyloid angiopathy, cerebral hemorrhage, human growth hormone, magnetic resonance imaging, neurosurgery, prions

## Abstract

Prion-like transmission of amyloid-ß through cadaveric dura, decades after neurosurgical procedures, has been hypothesized as an iatrogenic cause of cerebral amyloid angiopathy (CAA). We investigated new and previously described patients to assess the clinical profile, radiological features, and outcome of this presumed iatrogenic CAA-subtype (iCAA).

**Methods:**

Patients were collected from our prospective lobar hemorrhage and CAA database (n=251) with patients presenting to our hospital between 2008 and 2022. In addition, we identified patients with iCAA from 2 other Dutch CAA-expertise hospitals and performed a systematic literature-search for previously described patients. We classified patients according to the previously proposed diagnostic criteria for iCAA, assessed clinical and radiological disease features, and calculated intracerebral hemorrhage (ICH)-recurrence rates. We evaluated the spatial colocalization of cadaveric dura placement and CAA-associated magnetic resonance imaging markers.

**Results:**

We included 49 patients (74% men, mean age 43 years [range, 27–84]); 15 from our database (6% [95% CI, 3%–10%]; 45% of patients <55 years), 3 from the 2 other CAA-expertise hospitals, and 31 from the literature. We classified 43% (n=21; 1 newly identified patient) as probable and 57% (n=28) as possible iCAA. Patients presented with lobar ICH (57%), transient focal neurological episodes (12%), or seizures (8%). ICH-recurrence rate in the new patients (16/100 person-years [95% CI, 7–32], median follow-up 18 months) was lower than in the previously described patients (77/100 person-years [95% CI, 59–99], median follow-up 18 months). One patient had a 10 year interlude without ICH-recurrence. We identified no clear spatial relationship between dura placement and CAA-associated magnetic resonance imaging markers. During follow-up (median, 18 months), 20% of the patients developed transient focal neurological episodes and 20% cognitively declined.

**Conclusions:**

iCAA seems common in patients presenting with nonhereditary CAA under the age of 55. Clinical and radiological features are comparable with sCAA. After diagnosis, multiple ICH-recurrences but also long symptom-free intervals can occur. Harmonized registries are necessary to identify and understand this potentially underrecognized CAA-subtype.

**See related article, p 1224**

Cerebral amyloid angiopathy (CAA) classically presents in the elderly with intracerebral hemorrhage (ICH) and cognitive decline as a result of pathological changes in leptomeningeal and small cortical vessels. Impaired clearance of Aß (amyloid-ß) protein through the perivascular drainage systems and glymphatic pathway are considered as the most likely disease mechanisms.^1^

Nevertheless, new insights arose several years ago with reports of young patients developing CAA 2 to 3 decades after undergoing (neuro)surgical procedures during childhood. It was hypothesized that this suspected iatrogenic form of CAA (iCAA) was caused by prion-like transmission of Aß from cadaveric material used during these procedures. Proposed transmission routes were through cadaveric dural grafts (cadaveric dura) or embolization material,^2–7^ the use of cadaveric human growth hormone,^8–10^ or through surgical instruments.^11–13^ Recently, diagnostic criteria for iCAA were published, which help to standardize investigation of this new CAA-subtype.^14^

We aim to describe the frequency, disease course, and radiological features of iCAA by investigating a series of newly identified cases with suspected iCAA in the Netherlands and previously described iCAA cases in the literature.

## Methods

### Data Collection

We identified patients with potential iCAA from the following 3 sources: (1) the CAA-database of the Leiden University Medical Center (LUMC), (2) 2 other CAA expert centers in the Netherlands (the Radboud University Medical Centre and the University Medical Center Utrecht), and (3) the literature.

From our prospectively collected database at the LUMC, including all patients who presented at the (out)patient clinic between January 2008 and August 2022, with ICH, transient focal neurological episodes (TFNE), or cognitive decline, and radiological evidence of CAA on magnetic resonance imaging (MRI). Because deep hemorrhagic lesions are allowed in the diagnostic criteria for iCAA, we allowed presence of deep hemorrhages as long as the distribution was predominantly lobar.^14^ We reviewed all medical records of these patients to identify those who underwent neurosurgery or other (cardio)surgical procedures that might have involved cadaveric dura (see the Supplemental Material for time-frame and considered procedures). We retrieved information from additional patients with suspected iCAA from the Radboud University Medical Center and the University Medical Center Utrecht.

In addition, we systematically searched PUBMED, EMBASE, and Web of Science, to identify all patients with suspected iCAA in the literature published between database inception and August 2022. We used the search terms “Cerebral Amyloid Angiopathy” combined with “dura mater,” “cadaver,” “Lyodura,” “(neuro)surgery”, or “neurosurgical drains” (extra-ventricular and ventriculo-peritoneal drains) without language restrictions. Titles and abstracts were screened for relevance by one investigator (Dr Kaushik), excluding reports of patients with combined Creutzfeldt Jakob Disease and CAA. Data of duplicate patients were reported once.

This study was approved by the LUMC Medical Ethics Committee, which concluded that it did not fall under the medical research on human subjects act (non-WMO). This article follows the STROBE reporting guideline (Supplemental Material).^15^ The data that support the findings of this study are available from the corresponding author upon reasonable request.

### Classification and Assessment

Patients were classified using the previously proposed diagnostic criteria for iCAA (Table S1).^14^ The genetic-panel for new Dutch patients is presented in the Supplemental Material. The age criterium was not adjusted for the Boston criteria 2.0.^16^ Patients with neurosurgery after CAA-diagnosis were not considered.

For all patients, we collected information on demographics, genetic testing, diagnostic workup, and the surgery report. Information on clinical presentation including symptoms of CAA, such as lobar ICH, cognitive decline, or TFNEs, was retrieved from medical records (for newly identified patients) or from the previously published case-reports. Genetic testing is part of regular diagnostic work-up in young patients presenting to the LUMC, who are suspected of having CAA or in patients who have a family history of ICH or young-onset dementia. Information on seizures and related cortical swelling (abnormal cortical signal and gyral swelling on fluid-attenuated inversion recovery imaging) was collected, as this was previously reported in iCAA, possibly in the context of seizures.^14^

In the new patients, we prospectively rated the presence of CAA markers on MRI (macrobleeds and cerebral microbleed [CMB]), cortical superficial siderosis (focal if restricted to ≤3 sulci; disseminated if >3 sulci involved), convexity subarachnoid hemorrhage, centrum semiovale enlarged perivascular spaces (grade ≤2 if 0–20 visible; grade ≥3 if >20 centrum semiovale enlarged perivascular spaces [Supplemental Material]), white matter hyperintensities, and recent small subcortical infarcts) according to the Strive criteria, and assessed their progression over time.^17,18^ Number of recurrent symptomatic ICHs and clinical follow-up data from first presentation to last recorded visit was derived from medical records. Similarly, presence and progression of radiological CAA markers and clinical course were based on available reporting of the previously published patients.

### Statistics

We used descriptive statistics and calculated frequencies of patients with suspected iCAA including 95% CI in the LUMC CAA-database population, overall and for patients <55 years (young) and ≥55 separately. Radboud University Medical Center/University Medical Center Utrecht patients were nonsystematically collected and therefore, not included in the calculated frequency. An experienced neuroradiologist explored the spatial colocalization of CAA markers (hemorrhagic and nonhemorrhagic separately), and the laterality of cadaveric dura placement, through visual analysis. As signs of intracranial surgery remain visible on MRI, visual analysis was unblinded for graft-laterality. We calculated the rate of symptomatic ICH-recurrence/100 person-years and compared these for patients aged <55 and ≥55 years using Fisher exact test. Age-restricted sensitivity analysis for young cases (<55 years at presentation and <50 years separately) was done.

## Results

### Frequency and Classification of iCAA

#### Newly Identified Cases in the Netherlands

**Figure 1.**
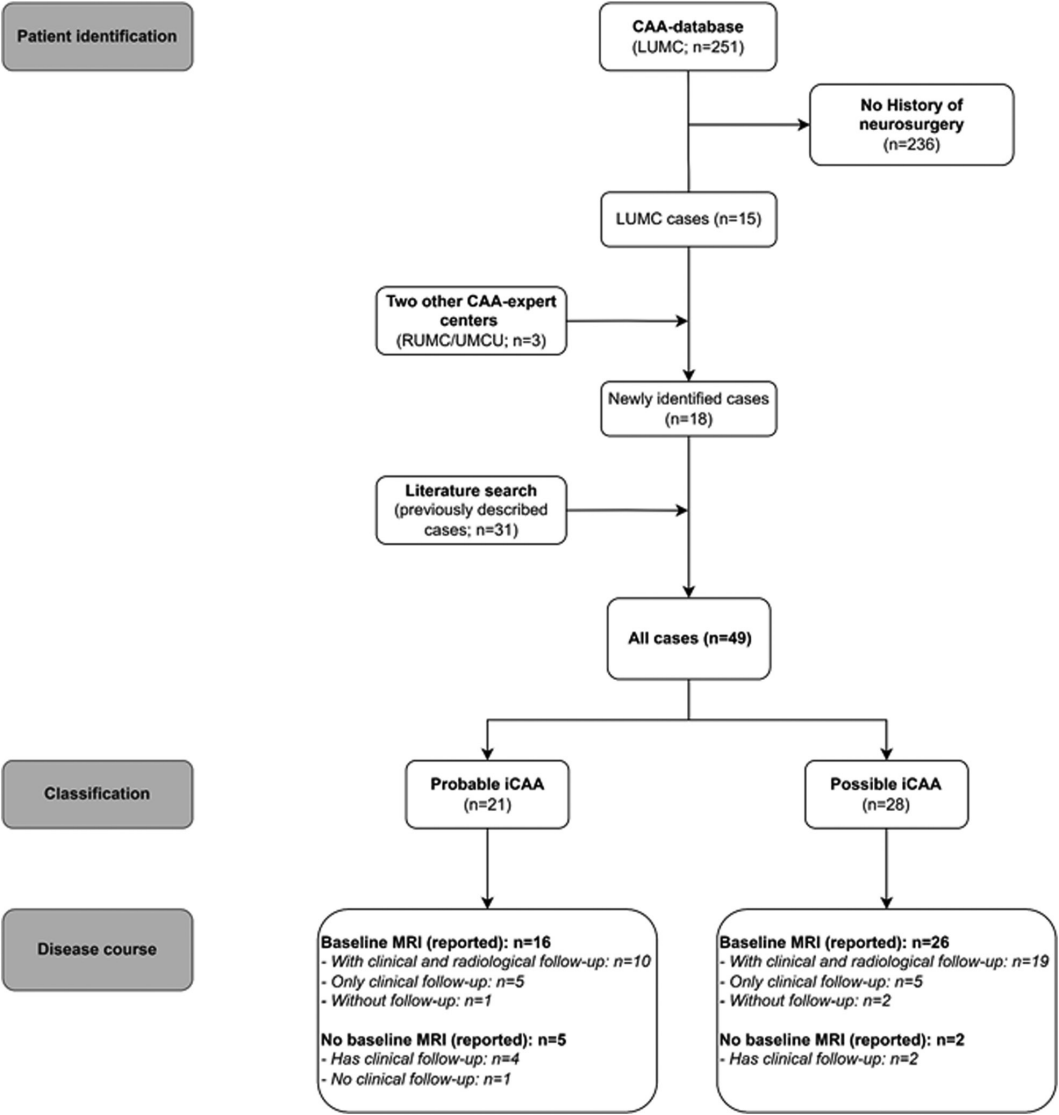
**Flowchart of patient identification, classification, and available disease course data.** CAA indicates cerebral amyloid angiopathy; iCAA, iatrogenic CAA; LUMC, Leiden University Medical Center; MRI, magnetic resonance imaging; RUMC, Radboud University Medical Center; and UMCU, University Medical Center Utrecht.

We identified 18 patients with neurosurgery in the Dutch Lyodura time-frame (Figure 1): 15/251 (6% [95% CI, 3%–10%]) patients with CAA from the LUMC database (9/20 [45%] young patients; 6/231 [3%] of patients ≥55 years of age at diagnosis), and 3 patients were from 2 other Dutch academic CAA-expertise hospitals. Three percent of LUMC patients aged ≥55 years who meet the modified Boston criteria, and 4% of those aged >50 years and who meet the Boston criteria 2.0, also qualified for the criteria for iCAA. Eight of the 18 (44%) newly identified patients had confirmed use of Lyodura and 10 had undergone relevant neurosurgical procedures (Table 1). In 8/10 patients with relevant neurosurgical procedures, use of Lyodura rather than nonbiological dura plastics was part of regular care for that procedure. No genetic-mutations were found for the *APP* (amyloid-beta precursor protein)-gene (tested n=12 [67%]) or in next-generation sequencing (tested n=12 [67%]; *PSEN* [presenilin]*1/2* and *PRNP* [prion protein gene] tested n=3). None of them had a family history of dementia, CAA, or ICH. Positron emission tomography was performed in 2/18 (11%) new patients: one with high Aß-uptake signal (C-labeled Pittsburgh B), and one in whom Alzheimer disease was excluded because there was no increased 18F-flutemetamol uptake in the gyrus cinguli posterior, precuneus, frontal lobe, parietal lobe temporal lobe, or basal ganglia. However, in the latter patient a diagnosis of CAA could neither be supported or excluded, since the scan was not performed to assess CAA and 18F-flutemetamol insufficiently binds to Aß in vessels. This, in combination with the limited resolution of this technique, limits the ability to distinguish uptake in neuritic plaques from uptake in the brain vasculature or the parenchyma. Lumbar puncture and histopathology were not available.

**Table 1.**
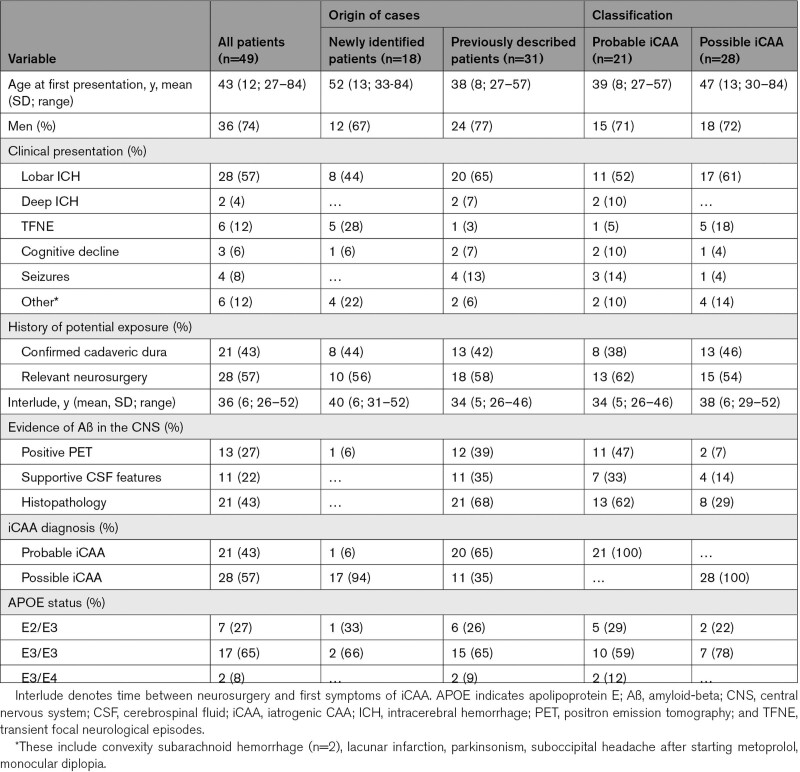
Demographic Characteristics of Suspected Iatrogenic CAA Patients

Seven (3%) of the LUMC patients had surgery prior to CAA diagnosis, but were insufficiently suggestive according to the criteria of probable or possible iCAA to be diagnosed as such.

#### Previously Described Patients in the Literature

We identified 31 patients from the literature, of whom 20 (65%) were classified as probable iCAA (8 confirmed use of Lyodura) and 11 as possible iCAA (5 confirmed use of Lyodura).^5–7,11–14,19–30^ Histopathology was available in 21/31 patients (20 brain biopsies and 1 postmortem examination) and confirmed the presence of CAA in all. Tau pathology was investigated in 6 and present in 3 (50%) patients. Presence of abnormal PrP (prionprotein) was investigated and excluded in 4 patients. No genetic-mutations were found for the APP-gene (tested n=25 [81%]), *PSEN1/2* genes (tested n=21 [68%]), or next-generation sequencing (tested n=3 [10%]). None of these cases had a family history of dementia, CAA, or ICH.

### (Neuro)surgical Interventions

Most frequent indications for surgery in the 21 patients with probable iCAA were traumatic brain injury (43%) and intracranial tumor resection (14%). The most frequent indication for surgery in the 28 patients with possible iCAA was traumatic brain injury (32%). All neurosurgical procedures of the 18 new cases were without procedural complication (Table S2). A ventriculo-peritoneal drain was placed in 8/18, while postoperative complications and drain placement was not reported in any of the reported patients in scientific literature.

### Clinical Features

Of all identified patients with iCAA, 36/49 (74%) were men. Mean age at presentation was 43 years (range 27–84). In total, 20/21 (95%) patients with probable iCAA and 21/28 (75%) patients with possible iCAA were diagnosed at age <55 years. Most frequent presentations were lobar ICH (57%, 1 patient with simultaneous thalamic ICH), TFNE (12%), and seizures (8%). Of all young patients, 25/41 (61%) presented with lobar ICH. In all patients with iCAA, mean time between the neurosurgery and clinical presentation was 36 years (SD, 6; range, 26–52 years).

### Radiological Features

**Figure 2.**
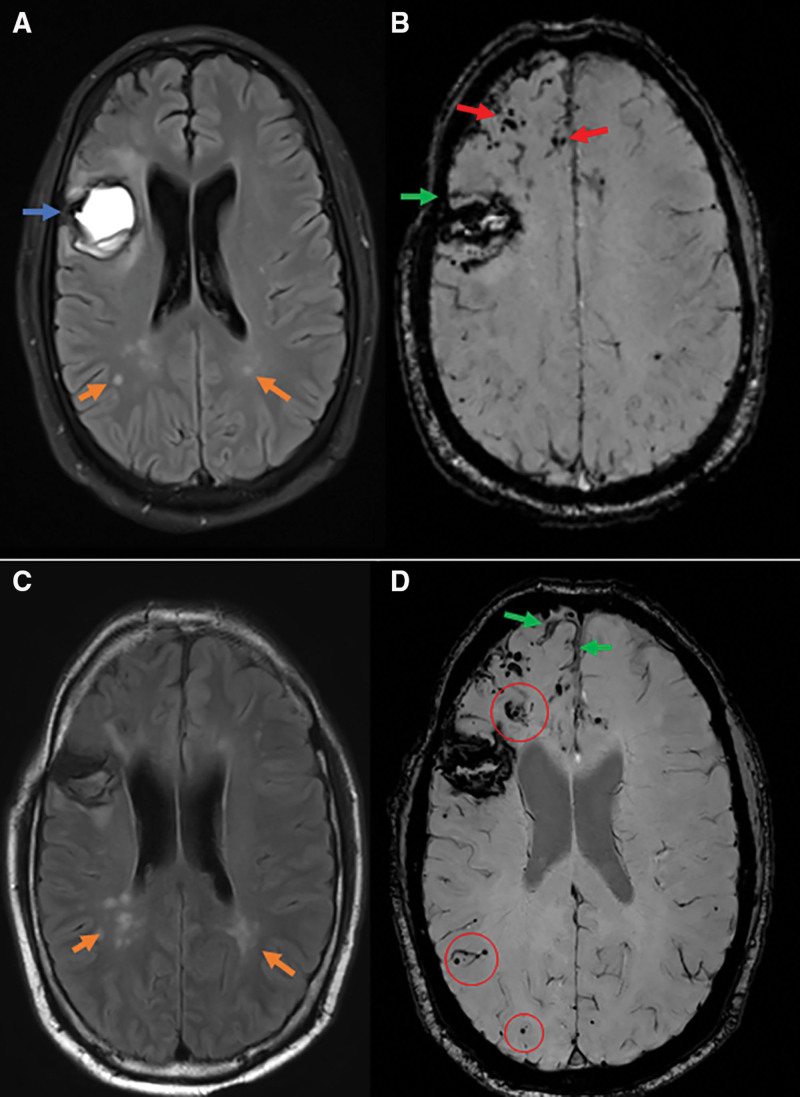
**Magnetic resonance imaging (MRI) findings and follow-up of one patient with possible iatrogenic cerebral amyloid angiopathy (CAA).** Forty-four-year-old man, with prior history of neurosurgery (1976) for an encephalocele with hydrocephalus, with confirmed use of Lyodura in the right frontal lobe, presenting 44 y later with acute aphasia due to a lobar intracerebral hemorrhage (ICH; **A**; fluid-attenuated inversion recovery imaging [FLAIR]; blue arrow) that was located in the proximity of the resection cavity. Baseline susceptibility weighted imaging (SWI; **B**) showed multiple cerebral microbleeds (CMB; red arrows) and cortical superficial siderosis (cSS; green arrow) in the vicinity of the ICH. Four mo after presentation he developed tonic-clonic seizures, no MRI was performed. Follow-up SWI after 11 mo (**D**) showed progression of cSS (green arrows) and new incident CMBs (red circles) in the right frontal, temporal, and both occipital lobes. Note that due to difference in angulation of slices between baseline and follow-up SWI only the frontal region can be compared on the images shown. Follow-up FLAIR-imaging showed increase of white matter hyperintensities in both parietal lobes (**A** denotes presentation, and **C** denotes follow-up; orange arrows).

Radiological data was available in 42/49 (86%) patients (Table 2). Cerebellar imaging was only available in all newly identified patients (3 Tesla MRI n=15, 1.5 Tesla MRI n=3). Radiological characteristics are presented as n (%) of available MRI reporting and sequences. At baseline, 26 (84%) patients had strictly lobar CMB or no reported deep CMB, 4 (13%) had concomitant deep CMB, and 1 had no CMB. Cortical superficial siderosis was observed in 19 (74%) of patients: 8 focal and 11 disseminated cortical superficial siderosis. Cerebellar CMBs were present in 8 (44%) patients. Furthermore, convexity subarachnoid hemorrhage was observed in 7 (44%) patients and 5 (28%) had recent small subcortical infarcts. Figure 2 and Figure S1 show the MRI findings and follow-up of 2 illustrative cases.

**Table 2.**
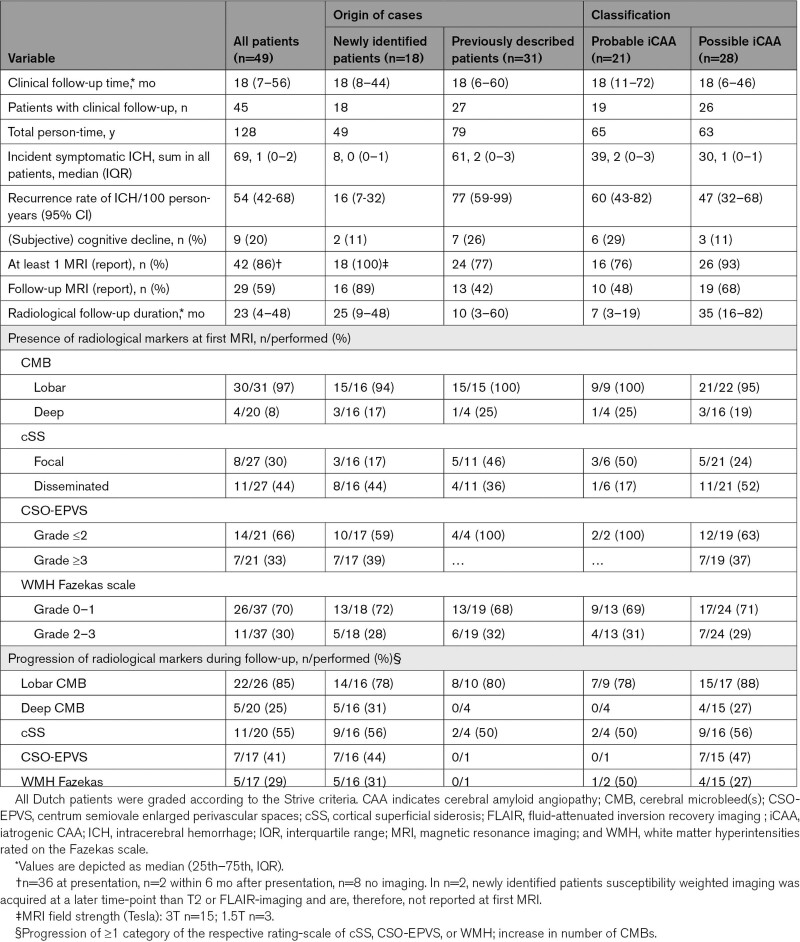
Disease Course of Suspected Iatrogenic CAA Patients

A possible association between the location of the radiological findings and the neurosurgical site was mentioned in one patient from the literature, in whom most CMBs were localized over the primary graft level.^25^ Similarly, in one newly identified patient hemorrhagic radiological markers were predominantly colocalized in the region of neurosurgery (Figure 2). However, no spatial relation was observed in any of the other patients (n=8 possible to assess, n=14 extra cranial-neurosurgeries, n=16 precise surgical site unknown) between hemorrhagic (including ICH) or nonhemorrhagic CAA MRI-markers and the surgical site or neurosurgical drain trajectory (Table S3).

### Clinical Outcome and Recurrent ICH Risk

Follow-up was after first presentation was available in 45 patients (not reported in 4 previously described patients). Median follow-up time was 18 months (interquartile range, 7–56; range, 1–146). During follow-up, in total 69 recurrent symptomatic ICHs occurred in 28 (62%) patients (median 1; interquartile range, 0–2). Five (11%) patients died due to ICH. Nine out of 45 (20%) patients experienced TFNEs, 9 (20%) cognitively declined, and 7 (16%) developed seizures. ICH-recurrence rate in all patients was 54/100 person-years (95% CI, 42–68); ICH-recurrence rate was 63 per 100 person-years in young patients (median follow-up 15 months, 104 person-years) versus 13 per 100 person-years in patients aged ≥55 years (median follow-up 21 months, 24 person-years; *P*=0.0004). The ICH-recurrence rate was substantially higher in the literature cases than in the new cases (77 versus 16 per 100 person-years; Table 2). Although most patients had recurrences at short-term follow-up, in one newly identified patient there was a 10-year interval without any clinical symptoms.

### Progression of Radiological Markers

Follow-up MRI was performed in 29/49 (59%) patients. The majority of patients had progression of CMBs (85%), cortical superficial siderosis (55%), or centrum semiovale enlarged perivascular spaces (41%) during a median radiological follow-up time of 23 months (interquartile range, 4–48). New recent small subcortical infarcts were observed in 4 (14%) patients. Twenty-seven new asymptomatic ICH were observed during follow-up in 20 patients (median 0; interquartile range, 0–1; max 4). Two patients experienced asymptomatic ICH-recurrence in the lobe of neurosurgery. During follow-up, CMBs and/or ICH in deep locations were present in 7/49 patients (14% [95% CI, 6%–26%]). Cortical swelling was reported in 2 literature cases: one with uncertain diagnosis of seizures, one 18 months after generalized tonic-clonic seizures.^6^ No newly identified patients had signs of cortical swelling which could be related to epilepsy.

### Age-Restricted Sensitivity Analysis

For the sensitivity analysis for cases presenting at age <55 years (n=41), we excluded 1 probable iCAA (with relevant neurosurgery; previously identified) and 7 patients with possible iCAA (n=1 confirmed cadaveric dura; all newly identified). Other than decreased estimate-precision of ICH-recurrence rates, no meaningful differences in disease features, clinical or radiological course were observed (not shown). Extending the sensitivity analysis to only cases aged <50 years (n=37), we excluded another probable case (confirmed cadaveric dura; previously identified) and 3 possible iCAA cases (n=1 confirmed cadaveric dura; n=2 newly identified). In this analysis, the interlude between neurosurgery and presentation was identical to the main analysis in the probable iCAA cases, but lowered in the possible iCAA cases (sensitivity: mean 26 [SD5]; range 29–45 years; main: 38 [6], 29–52 years). No further meaningful differences were observed (Tables S4 and S5).

## Discussion

In our study, nearly half of young patients with CAA were suspected of having iCAA. Of our patients diagnosed as probable CAA according to the modified Boston criteria, 3% might have iatrogenic cause of CAA (4% according to the Boston Criteria 2.0). Similar to sporadic CAA, the majority of patients with suspected iCAA presented with ICH, cognitive decline, or TFNEs. In almost all patients hemorrhagic markers on MRI were strictly lobar, although deeply located microbleeds and/or ICH were present in 14% of patients. Our data suggest that iCAA can have multiple ICH-recurrences but also long symptom-free intervals.

Increasing evidence from animal studies suggests that prion-like transmission of Aß might be the disease mechanism in iCAA. Exogenous Aß-seeds introduced in mice have shown to cause vascular depositions of Aß more than depositions in the brain parenchyma.^31^ This might be related to structural differences in Aß-40 and Aß-42. Accumulating evidence for the propagation of Aß across parenchymal tissue has been published previously.^32,33^ Nevertheless, the route of Aß-seeding through the brain remains unclear. We did not observe an apparent spatial colocalization of CAA MRI-markers, and the laterality of cadaveric dura placement. However, the occurrence of hemorrhages in deep locations could suggest migration of Aß from the surgical site. Possible propagation routes include intra- or extra-cellular spreading throughout the glymphatic or hematogenic systems, or the subarachnoid space.

An alternative hypothesis is that neurosurgery itself could affect brain clearance of Aß. Neurosurgery (including placement of ventricular drains) could result in alterations of glymphatic fluxes and of the hydrostatic and osmotic pressure gradients, thereby impairing glymphatic functioning locally and further downstream.^34,35^ However, the high frequency of exposure to cadaveric material in patients with iCAA, along with the similarity of interlude between neurosurgery and CAA-onset to that in iatrogenic Creutzfeldt-Jakob disease challenges this hypothesis.

The number of men in our cohort was higher than the number of women. One explanation for this finding could be that men in general have traumatic brain injury more often and, therefore, a higher risk for a neurosurgical procedure in which Lyodura could have been used. However, there were no sex-differences in relative risk of receiving any of the neurosurgical procedures including procedures related to traumatic brain injury (Table S2). Another hypothetical explanation for the high number of men in our study is underrepresentation of women due to a protective effect of estrogen against Aβ-accumulation or inflammation.^36^ However, given the current limited state of knowledge on the role of estrogen in CAA, the observed high proportion of men might probably be best explained as random variation.

It remains uncertain how many patients who are exposed to cadaveric dura mater may develop iCAA, and how many patients with iCAA remain undiagnosed. There is no registration of the prior use of cadaveric dural grafts in the Netherlands. It is probable that the clinicians’ awareness for uncommon causes is less in older patients. Therefore, we might have underestimated prevalence of dural grafts in patients aged ≥55 years. Similarly, due to the age-criterion in the Boston criteria, young iCAA (<55 years in modified Boston criteria; <50 years in Boston criteria 2.0) might be underreported because CAA was not considered as a diagnosis. Lyodura was used in a wide range of surgical procedures in 1968 to 1990 after which it was banned. Because we did not systematically interview patients about their surgical history or use of cadaveric hormones, possible cases with Aß-seeding through the hematogenic or lymphoid systems might have been missed. Some studies have estimated total use of >2200 grafts in Australia, to <400 grafts in the United States, or 20 000 grafts yearly during the 13-year usage period in Japan.^37,38^ These numbers imply that the population at risk for developing iCAA over the world might be substantial and that more cases might be identified in the upcoming years.

It is unknown whether the brains of younger persons are more susceptible for developing iCAA, or whether time after neurosurgery is the most important factor for developing this disease subtype. The interlude between surgery and CAA presentation is strikingly comparable to the interlude in Dutch-type hereditary CAA between cerebral Aß accumulation and the occurrence of the first symptoms. Patients with this autosomal dominant hereditary form of CAA usually develop their first ICH at the age of 50 to 55, whereas abnormalities in Aß levels in the CSF can already been detected in presymptomatic mutation carriers from the age of 25.^39,40^ These data suggest that development and progression of iCAA is a rather slow process.

The previously proposed diagnostic criteria recognize the relative certainty of evidence of CAA and potential exposure to exogeneous Aß in patients with iCAA. This ensures a standardized diagnosis for patients with probable iCAA. Currently, the diagnostic performance of these criteria is unknown, as they have not yet been validated as and no diagnostic reference standard yet exists for iCAA. An important drawback of this classification is the heterogeneity in the possible iCAA category and the lack of restriction to the period when cadaveric material was used. For example, patients with confirmed use of Lyodura and exclusion of genetic causes through extensive next-generation sequencing, are more likely to have iCAA than those who had neurosurgery outside of the Lyodura-era. Also, the underlying pathophysiological hypothesis of prion-like spreading implies that iCAA is a calendar time-bound, and not age-bound disease. As the Lyodura-era differs per country, and as an age-cutoff does not support pathophysiological reasoning, a future perspective should be to add an interlude-criterion to the classification. A further differentiation of high and low suspect possible iCAA patients should be considered when more cases have been collected. In addition, a more precise estimation of ICH-recurrence is required since the recurrence rate derived from the literature is substantially higher than we observed in our new patients, who were in majority derived from a prospective database with regular clinical follow-up. This is likely due to selection and publication bias because clinicians are more likely to notice patients with recurrent hemorrhage than those with single lobar ICH. Besides, the prognosis of iCAA does not always seem detrimental, as was shown in one case who lived a decade without symptoms before having a recurrent ICH. Nonetheless, the observed recurrence rate in newly reported patients remains higher compared with sCAA and seems similar to that of hereditary Dutch type CAA (14–28 ICH/100 person-years).^40^

Our study has limitations. Histopathology, diagnostic positron emission tomography-CT, and lumbar puncture are scarcely acquired to support the diagnosis of CAA in the Netherlands. Currently, there is insufficient consensus on the diagnostic value of positron emission tomograph-CT and CSF, for it to be used in clinical practice. This is illustrated by their absence in the Boston criteria 2.0 and restricts the clinical applicability of the diagnostic criteria for iCAA. Similarly, due to its low prevalence, *PSEN1/2*-mutations are not regularly assessed in next-generation sequencing. Unfortunately, these findings were unavailable in the majority of our patients, which might have caused differential misclassification. Further, CAA-histopathology was only present in one of the newly identified patients and in 62% of all patients with probable iCAA. This may have led to differential misclassification of non-CAA patients as iCAA. To be consistent with the previously proposed criteria for iCAA, we did not adapt the age-criterium to the Boston criteria 2.0. By definition, this did not result in misclassification of probable iCAA in our study. The 4 patients with possible iCAA aged 50 to 55 all had either confirmed cadaveric material use, or met the fourth or fifth diagnostic criterium. Finally, radiological markers were scarcely reported in the literature. Strengths of our study are the relatively large number of new patients with suspected iCAA, combined with the reported patients in the literature, which enabled research into frequencies, clinical features and outcome.

An important step to increase clinical understanding, is to further explore this association by systematically asking all (young) patients with CAA in clinical care for a history of neurosurgery, or other procedures in which cadaveric dura material could have been used. Histopathological research of cadaveric grafts on transmission of Aß and postmortem examination of patients with probable iCAA seems highly necessary to further understand the underlying mechanisms of this CAA subtype. National and international registries can aid us to do so.

## Article Information

### Sources of Funding

None.

### Disclosures

Dr Wermer reports independent support from the Netherlands Organization for Scientific Research (NWO VIDI grant 9171337), the Dutch Research Council (NWO memorable BIONIC [Biomarkers for Cognitive Impairment due to Cerebral Amyloid Angiopathy] 733050822), the Dutch Heart Foundation (Clinical Established Investigator grant 2016T86), the Dutch Brain Foundation and the Dutch cerebral amyloid angiopathy (CAA) foundation. Dr Terwindt reports independent support from the Dutch Research Council (Nederlandse Organisatie voor Wetenschappelijk Onderzoek memorable BIONIC 733050822), the Dutch Heart Foundation, the Dutch Brain Foundation and the Dutch CAA foundation. Dr Schreuder reports independent support from the Dutch Heart Foundation (Senior Clinical Scientist grant 2019T060), the Dutch Heart Foundation and the Swedisch Orphan Biovitrum AB. Dr Klijn reports disclosures all unrelated to this study: support from the Netherlands Cardiovascular Research Initiative, which is supported by the Dutch Heart Foundation, CVON2015-01: CONTRAST (Collaboration for New Treatments of Acute Stroke), and the support of the Brain Foundation Netherlands (HA2015.01.06). The collaboration project is additionally financed by the Ministry of Economic Affairs by means of the public-private partnerships allowance made available by the Top Sector Life Sciences & Health to stimulate public-private partnerships (LSHM17016). This work was funded in part through unrestricted funding by Stryker, Medtronic, and Cerenovus; the funding sources were not involved in study design, monitoring, data collection, statistical analyses, interpretation of results, or article writing. Radboud University Medical Center and Erasmus MC received additional unrestricted funding on behalf of CONTRAST, for the execution of the Dutch Intracerebral Hemorrhage (ICH) Surgery Trial pilot study from Penumbra Inc. For the Dutch ICH Surgery Trial, they also received a grant from ZonMw/Promising care (grant 80- 86200-08-25001). The other authors report no conflicts.

### Supplemental Material

Supplemental Methods

Tables S1–S5

References 37,41

## Supplementary Material
