# Supplementary material for: Iatrogenic Cerebral Amyloid Angiopathy Post Neurosurgery: Frequency, Clinical Profile, Radiological Features, and Outcome
Source: Stroke. 2023 Apr 10;54(5):1214–23. doi: 10.1161/STROKEAHA.122.041690 (PMC10121246; doi:10.1161/STROKEAHA.122.041690)
Supplement: Supplementary file 1 [file str-54-1214-s001.pdf]

SUPPLEMENTAL MATERIAL

Supplemental Methods

Procedures considered for relevant (neuro)surgical procedures.

In the Netherlands, lyophilized irradiated cadaveric dura named Lyodura was used from 1968 to approximately 1990 when it was associated with the development of iatrogenic Creutzfeldt Jakob Disease (iCJD). These grafts were mostly used for neurosurgical procedures, including sealing dural defects after tumor resection, skull fractures, posterior fossa surgery, and in spinal repairs for spina bifida, or iatrogenic dural defects in the spine.[41] Other surgical procedures in which it was used include pericardium repairs and abdominal hernias.[37] Abdominal hernia repairs were not considered as relevant surgical procedures in the current study.

Next-generation genetic sequencing performed in Dutch patients

Genes tested in panel: *NOTCH3, APP, TREX1, HTRA1, ABCD1, AUH, CBS, CLCN2, COL4A1, COL4A2, CSF1R, CST3, CYP27A1, CSTA, DARS2, GBE1, GFAP, GLA, GSN, ITM2B, LMNB1, MMACHC, TREM2, TTR, TYMP, TYROBP*

In n=3 patients (all presenting after publication of the proposed diagnostic criteria for iCAA) *PSEN1, PSEN2* and *PRNP* were tested.

Rating of enlarged perivascular spaces (EPVS) in the centrum semi-ovale

4-points rating scale: 0 if no EPVS visible, 1 if <10 EPVS, 2 if 11-20 EPVS, 3 if 21-40 EPVS, and 4 if >40 EPVS visible.

Table S1: Summarized diagnostic criteria for iatrogenic CAA

Criterium	Probable iCAA	Possible iCAA
1) Age of onset <55 years	Preferred	Required for consideration
2) History of potential exposure (one or more)<br>• Procedure/treatment using cadaveric human CNS tissues<br>• Relevant neurosurgical procedure (brain, spinal cord, posterior eye)	Required	Required for consideration
3) Clinical and radiological features of CAA, with predominantly lobar distributed cerebral microbleeds	Required	Required for consideration
4) Evidence of amyloid-beta accumulation in the CNS<br>• Positive amyloid PET-scan<br>• Supportive CSF-features<br>• Brain biopsy demonstrating vascular Aß-deposition without significant inflammation	Required	
5) Exclusion of genetic causes of Aß-CNS disease<br>• APP, PSEN1/2<br>• Next-generation sequencing for non-Aß CAA mutations	Required	

See original article for full criteria[14]; Possible iCAA can be considered if criteria 1-3 are met

Table S2: (Neuro)surgical interventions in suspected iCAA patients

Intervention	All patients (n= 49)	Origin	Classification	
		New Dutch patients (n= 18)	Probable iCAA (n= 21)	Possible iCAA (n= 28)
Traumatic brain injury, n(%)	18 (37)	3 (17)	9 (43)	9 (32)
Skull fracture		*1*	*2*	*4*
SDH/EDH evacuation		-	*3*	*2*
Not further specified		*2*	*4*	*3*
Tumor resection, n(%)	11 (22)	6 (33)	3 (14)	8 (29)
Astrocytoma		*5*	-	*7*
Meningioma		*1*	*1*	*1*
Eosinophilic granuloma		-	*1*	-
Medulla blastoma		-	*1*	-
Spina bifida, n(%)	6 (12)	4 (22)	1 (5)	5 (18)
Other resections, n(%)	5 (10)	2 (11)	3 (14)	2 (7)
Cerebellar cyst§		*1*	-	*1*
Choroid plexus papilloma		-	*1*	-
Nasopharyngeal angiofibroma		-	*1*	-
Parotid cavernous hemangioma		-	*1*	-
Subarachnoid cyst		*1*	-	*1*
Other procedures, n(%)	9 (18)	3 (17)	5 (24)	4 (14)
Encephalo-/meningocele repair		*1*	*2*	1
Hernia nucleus pulposi		*2*	-	2
Syringomyelia		-	*1*	-
Repair of the atrial septum (cardiac) †		-	-	1
Embolisation of postauricular AVM		-	*1*	-
Embolisation of haemangioma		-	*1*	-

AVM arterio-venous malformation; EDH indicates Epidural hematoma; iCAA, iatrogenic CAA; SDH, Subdural hematoma
§ One of the new Dutch probable iCAA patients might have undergone radiotherapy directly after extirpation of a cerebellar cyst, though this is undocumented and was not a regular procedure
† In one previously described patient cadaveric dura mater was used during cardiac surgery; the LUMC database was screened for cardiac procedures involving cadaveric dura mater, but no new patients were identified.

Number of men in all patients (n=36 men, n=13 women) undergoing intervention: traumatic brain injury n=14, tumor resection n=8, spina bifida n=3, other resections n=4, other procedures n=7

Table S3: visual analysis for spatial co-localization between MRI-markers and surgical site

	Assessment		Spatial co-localization present, n		ICH
**Neurosurgical Site**	Possible (n=8)	Not possible (n=41)	Hemorrhagic lesions (n=2)†	Ischemic lesions (n=0)‡	Same side as surgery (n=5)
Cerebrum					
Left	2	3	-	-	1
Right	5	6	2	-	4
Both sides	-	1	-	-	-
Brainstem	1	1	-	-	-
Per definition not possible*	-	14	-	-	-
Not reported	-	16	-	-	-

* Spatial co-localization of intracerebral MRI-markers to the surgical site is per definition not possible in extra-cranial (including spine) surgeries.

† Cerebral microbleeds, cortical superficial siderosis, or convexity subarachnoid hemorrhage.

‡ White matter hyperintensities or infarctions

Similar analysis for co-localization of MRI-markers and drain-trajectory was done, where no co-localization was observed in the same number of assessable cases.

Table S4: Demographics of suspected iatrogenic CAA patients, aged <50 years at presentation

		**Classification restricted to <50 years**	
**Variable**	All cases <50 years (n= 37)	Probable iCAA (n=19)	Possible iCAA (n=18)
Age at first presentation, years, mean(SD; range)	38 (6)	37 (7)	39 (5)
Men (%)	29 (78)	14 (74)	3 (17)
Clinical presentation (%)			
Lobar ICH	23 (62)	10 (53)	13 (72)
Deep ICH	1 (3)	1 (5)	-
TFNE	3 (8)	1 (5)	2 (11)
Cognitive decline	3 (8)	2 (11)	1 (6)
Seizures	4 (11)	3 (16)	1 (6)
Other	3 (8)	2 (11)	1 (6)
History of potential exposure (%)			
Confirmed cadaveric dura	18 (49)	7 (37)	11 (61)
Relevant neurosurgery	19 (51)	12 (63)	7 (39)
Interlude, years (mean, SD; range)	35 (5; 26-46)	34 (5; 26-46)	26 (5; 29-45)
Evidence of Aß in the CNS (%)			
Positive PET	11 (30)	10 (53)	1 (6)
Supportive CSF features	9 (24)	6 (32)	3 (17)
Histopathology	20 (54)	12 (63)	8 (44)
APOE status (%)			
E2/E3	6 (29)	5 (33)	1 (17)
E3/E3	13 (62)	8 (53)	5 (83)
E3/E4	2 (10)	2 (13)	-

APOE, apolipoprotein E; Aß, Amyloid-beta; iCAA, iatrogenic CAA; CNS, central nervous system; CSF, cerebrospinal fluid; ICH, intracerebral hemorrhage; Interlude, time between neurosurgery and first symptoms of iCAA; TFNE, transient focal neurological episodes.

Table S5: Disease course of suspected iatrogenic CAA patients, aged <50 years at presentation

Variable	All cases <50 years (n=37)	Classification restricted to <50 years: Probable iCAA (n=19)	Possible iCAA (n=18)
Clinical follow-up time,* months	18 (10-60)	34 (12-73)	13 (6-43)
Patients with clinical follow-up, n	33	17	16
Total person-time, years	97	65	32
Incident symptomatic ICH, sum in all patients, median (IQR)	63, 1 (0-3)	39, 2 (1-3)	24, 1 (0-2)
Recurrence rate of ICH/100 person-years (95%CI)	65 (50-83)	60 (43-82)	75 (48-112)
(Subjective) cognitive decline, n(%)	7 (21)	6 (32)	1 (6)
At least 1 MRI (report), n(%)	32 (87)	15 (79)	17 (94)
Follow-up MRI (report), n(%)	20 (54)	9 (47)	11 (61)
Radiological follow-up duration, * months	18 (4-68)	8 (3-26)	48 (14-107)
Presence of radiological markers at first MRI, n/performed (%)			
CMB			
Lobar	22/23 (96)	9/9 (100)	13/14 (93)
Deep	3/12 (25)	1/4 (25)	2/8 (25)
cSS			
Focal	6/19 (32)	3/6 (50)	3/13 (23)
Disseminated	7/19 (37)	1/6 (17)	6/13 (46)
CSO-EPVS			
Grade ≤2	9/13 (69)	2/2 (100)	7/11 (64)
Grade ≥3	4/13 (31)	-	4/11 (36)
WMH Fazekas scale			
Grade 0 – 1	20/27 (74)	8/12 (67)	12/15 (80)
Grade 2 – 3	7/27 (26)	4/12 (33)	3/15 (20)
Progression of radiological markers during follow-up, n/performed (%)§			
Lobar CMB	15/17 (88)	6/8 (75)	9/9 (100)
Deep CMB	5/12 (42)	1/5 (20)	4/7 (57)
cSS	7/12 (58)	2/4 (50)	5/8 (63)
CSO-EPVS	2/9 (22)	-	2/7 (29)
WMH Fazekas	2/9 (22)	1/2 (50)	1/7 (14)

CAA, Cerebral Amyloid Angiopathy; iCAA, iatrogenic CAA; ICH, intracerebral hemorrhage; CMB, cerebral microbleed(s); CSO-EPVS, centrum semi-ovale enlarged perivascular spaces; cSS, cortical superficial siderosis; WMH, white matter hyperintensities rated on the Fazekas scale; MRI, magnetic resonance imaging. All Dutch patients were graded according to the Strive criteria.
* Values are depicted as median (25th-75th interquartile range).
§ Progression of ≥1 category of the respective rating-scale of cSS, CSO-EPVS or WMH; increase in number of CMBs

Figure S1: MRI findings and follow-up of another patient with possible iatrogenic CAA

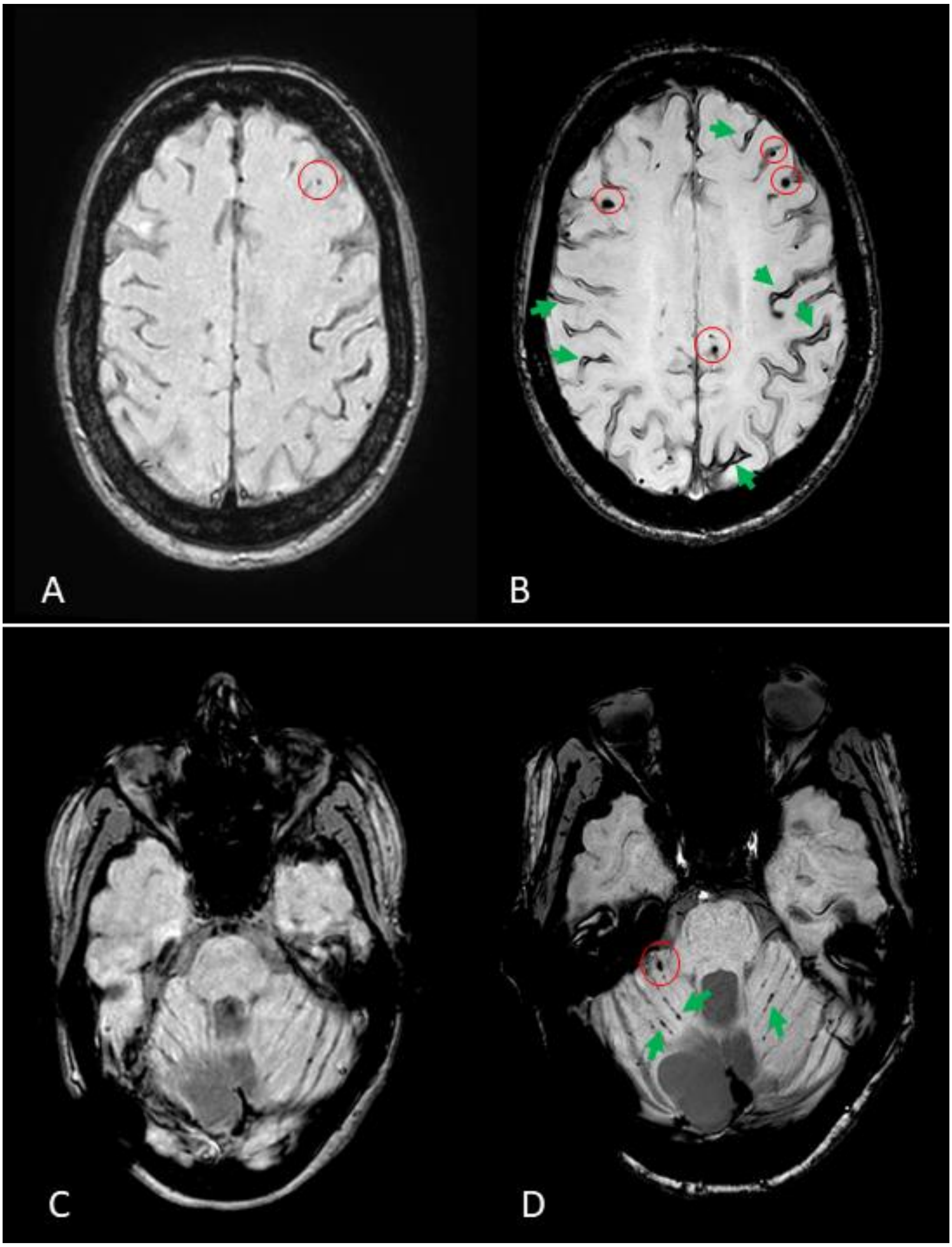

39-year-old woman in whom a left cerebellar astrocytoma was resected in 1986 (Lyodura unconfirmed but part of regular care for posterior fossa resections in the Netherlands), presenting after 34 years with coordination difficulties of the left hand and numbness of the left cheek, due to TFNEs. MRI at presentation showed multiple lobar CMBs (only one CMB is visible on image A; SWI; red circle), a macrobleed at the right frontal lobe, and cSAH (both not shown). Additionally, new supra- and infratentorial cSS (green arrows) and CMB (red circles) were observed on SWI (A,C: presentation; B,D: follow-up after 2 months). Four months later, there was no further clinical or radiological progression, images not shown.
